# Siderocalin/Lcn2/NGAL/24p3 Does Not Drive Apoptosis Through Gentisic Acid Mediated Iron Withdrawal in Hematopoietic Cell Lines

**DOI:** 10.1371/journal.pone.0043696

**Published:** 2012-08-21

**Authors:** Colin Correnti, Vera Richardson, Allyson K. Sia, Ashok D. Bandaranayake, Mario Ruiz, Yohan Suryo Rahmanto, Žaklina Kovačević, Matthew C. Clifton, Margaret A. Holmes, Brett K. Kaiser, Jonathan Barasch, Kenneth N. Raymond, Des R. Richardson, Roland K. Strong

**Affiliations:** 1 Division of Basic Sciences, Fred Hutchinson Cancer Research Center, Seattle, Washington, United States of America; 2 Iron Metabolism and Chelation Program, Discipline of Pathology and Bosch Institute, University of Sydney, NSW, Australia; 3 Department of Chemistry, University of California, Berkeley, California, United States of America; 4 Department of Immunology, University of Washington, Seattle, Washington, United States of America; 5 Instituto de Biología y Genética Molecular, Universidad de Valladolid, UVa-CSIC, Valladolid, Spain; 6 Emerald Biostructures, Bainbridge Island, Washington, United States of America; 7 College of Physicians and Surgeons of Columbia University, New York, New York, United States of America; 8 Seattle Structural Genomics Center for Infectious Diseases (SSGCID), Washington, United States of America; Roswell Park Cancer Institute, United States of America

## Abstract

Siderocalin (also lipocalin 2, NGAL or 24p3) binds iron as complexes with specific siderophores, which are low molecular weight, ferric ion-specific chelators. In innate immunity, siderocalin slows the growth of infecting bacteria by sequestering bacterial ferric siderophores. Siderocalin also binds simple catechols, which can serve as siderophores in the damaged urinary tract. Siderocalin has also been proposed to alter cellular iron trafficking, for instance, driving apoptosis through iron efflux via BOCT. An endogenous siderophore composed of gentisic acid (2,5-dihydroxybenzoic acid) substituents was proposed to mediate cellular efflux. However, binding studies reported herein contradict the proposal that gentisic acid forms high-affinity ternary complexes with siderocalin and iron, or that gentisic acid can serve as an endogenous siderophore at neutral pH. We also demonstrate that siderocalin does not induce cellular iron efflux or stimulate apoptosis, questioning the role siderocalin plays in modulating iron metabolism.

## Introduction

**Figure 1:**
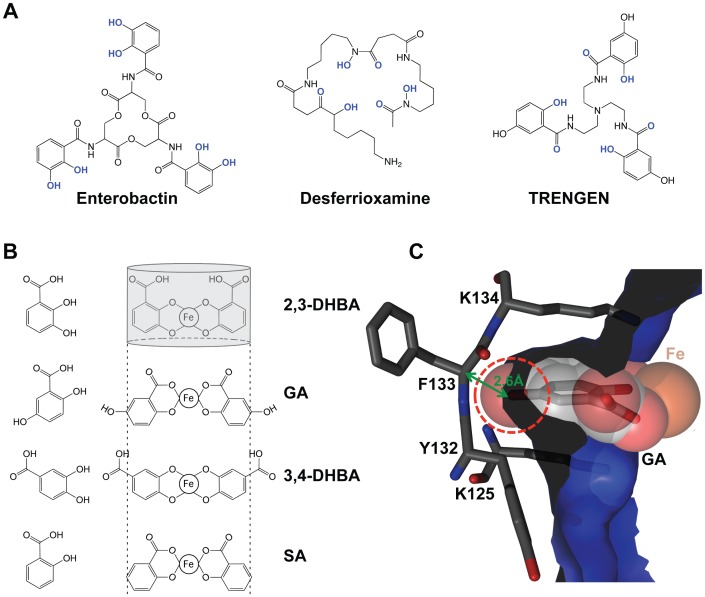
Steric clashes imposed by the Scn calyx preclude binding of ferric SA and GA complexes. (A) Hexadentate siderophore structures are shown with iron liganding atoms colored blue. (B) Structures of 2,3-DHBA, GA (2,5-DHBA), 3,4-DHBA and SA (2-hydroxybenzoic acid) are shown in the left column and complexes with iron in the center column (only two of three bidentate groups are shown for clarity). The Scn calyx is represented at top by a gray cylinder and the size constraint imposed by the calyx diameter is represented by dashed lines, schematically showing clashes with all iron complexes except 2,3-DHBA. (C) A section of the Scn/carboxymycobactin complex structure (PDB accession code 1X89) showing a GA moiety superimposed on the phenolate ring of carboxymycobactin. The steric clash of the 5-OH is indicated by penetrating the molecular surface of Scn (dashed red circle) and the short distance to neighboring atoms (green line).

Siderophores are low molecular weight, ferric i`on-specific chelators that some bacteria use to acquire iron [1]. The mammalian antibacterial protein siderocalin (Scn), also known as lipocalin 2 (Lcn2), neutrophil gelatinase-associated lipocalin (NGAL) or 24p3, functions by sequestering iron as bacterial siderophore complexes [2], [3]. Scn tightly binds a variety of bacterial siderophores including many catechol-based compounds from enteric bacteria, such as enterobactin (Ent; equilibrium dissociation constant (*K_D_*)  = 0.4±0.1 nM), but does not bind many hydroxamate-based siderophores, such as desferrioxamine (DFO; Figure 1A) [2]–[5]. The importance of Scn in antibacterial defense was demonstrated with Scn knock-out mice, which are profoundly susceptible to bacterial infections [2], [6]. Bacterial siderophores with modifications that ablate binding to Scn, so-called ‘stealth’ siderophores, allow pathogens to evade the Scn defense, permitting acquisition of iron during infection [2], [4], [5], [7]–[9].

Scn has also been implicated in cellular processes unrelated to antibacterial activities, including apoptosis and differentiation, reviewed in [10], [11]. Scn is observed in serum and urine in sterile kidney diseases and has been shown to be internalized by proximal tubule cells potentially after binding to the megalin receptor complex, leading to iron release from the protein [12], [13]. In these contexts, Scn enters endosomal compartments via the megalin receptor and passage through these low pH intracellular compartments correlates with iron release. To provide a rationale for its pleiotropic affects on apoptosis, siderophore-free Scn (apo-Scn) was reported to be secreted in response to cytokine withdrawal or tumorigenesis and internalized by a receptor-mediated process to alternately sequester and export intracellular iron, driving apoptosis through autocrine, paracrine, or exocrine mechanisms [14], [15]. This latter hypothesis was based on observations that: *i*) Scn transcription was maximally induced in murine hematopoietic cell lines undergoing IL-3 withdrawal-induced apoptosis; *ii*) conditioned medium from apoptotic cells containing secreted Scn, or addition of exogenous Scn, could induce apoptosis in susceptible cells even in the presence of IL-3; and *iii*) ectopic Scn expression conferred on cells the ability to import or export iron, in the latter case driving apoptosis by depletion of this essential nutrient [14], [15]. Internalization of Scn was shown to be mediated by a novel receptor, brain-type organic cation transporter (BOCT; also SLC22A17 or 24p3R), enabling access of apo-Scn to crucial intracellular iron pools vital for metabolism and proliferation [15].

Iron transport by Scn requires the presence of a siderophore, since Scn has no measurable affinity for iron alone [3]. Bacterial catecholate siderophores, like Ent and its substituent, 2,3-dihydroxybenzoic acid (2,3-DHBA; Figure 1B), are unlikely to fulfill the requirements of an iron delivery pathway because iron is not released from Scn/siderophore/iron complexes (holo-Scn) until acidification below pH 4, which is not readily achieved in most cellular compartments, such as endocytic vesicles [7]. Two candidate endogenous siderophores have been proposed: *i*) simple catechols, including catechol itself (1,2-dihydroxybenzene), mediating iron delivery [13], and *ii*) compounds that include gentisic acid (GA; 2,5-dihydroxybenzoic acid) substituents mediating cellular iron efflux [16]. Bao and coworkers reported that free catechol binds poorly to Scn (*K_D_* = 0.20±0.06 µM), but catechol/iron complexes bind tightly (*K_D1_* = 2.1±0.5 nM/*K_D_*_2_ = 0.4±0.2 nM), and that: *i*) catechol can mediate iron transport in the proximal kidney through Scn complexes potentially by the megalin receptor complex; *ii*) iron from Scn/catechol complexes is released at pHs below 6; and *iii*) Fe(catechol)_x_ can be directly visualized by X-ray crystallography bound in the Scn ligand-binding site or ‘calyx’. Devireddy and coworkers reported that: *i*) GA can be isolated from conditioned media; *ii*) binds to Scn tightly in the absence of iron (*K_D_* = 12 nM); *iii*) supports iron transport by Scn *in vitro*; and *iv*) is synthesized endogenously by a cytosolic type II *R*-β-hydroxybutyrate dehydrogenase (DHRS6) also known as BDH2.

Identification of GA alone or as a substituent of siderophores enabling Scn-mediated iron transport was surprising as GA and GA-based siderophores obligately interact with iron in a manner (salicylate-mode) that, either alone, but especially in combination with 5-OH groups, precludes binding to Scn (Figures 1B and 1C). In agreement with this prediction, we show here that both GA and a synthetic *tris*-GA analog (TRENGEN; Figures 1A and S1) bind weakly to Scn either in the presence or absence of iron. Like salicylic acid (SA; Figure 1B), we also show that GA on its own does not efficiently form iron complexes at neutral pH. These results show that GA does not meet the necessary biochemical criteria required for an endogenous siderophore or siderophore substituent enabling Scn-mediated iron transport.

Due to our failure to confirm the ability of GA to serve as a siderophore or to bind to Scn, we then also re-examined the reported roles of Scn in iron transport and apoptosis. In contrast to previous results using identical methodologies [14], [15], we demonstrated that HeLa cells ectopically expressing BOCT did not induce cellular iron efflux *via* Scn. Moreover, we showed Scn did not drive apoptosis in hematopoietic cell lines (FL5.12 and 32D.3) reported to be susceptible to this protein, even when Scn was added at levels exceeding those used previously by 200-fold. We also generated stable transductants, secreting high levels of Scn, in 32D.3 and FL5.12 cells without decreasing viability. Finally, we were unable to detect Scn protein secreted from FL5.12 or 32D.3 cells undergoing IL-3 withdrawal-induced apoptosis. We conclude: *i*) GA cannot bind Scn or serve as a siderophore under physiological conditions; *ii*) Scn does not participate in iron efflux mediated by interactions with BOCT in HeLa cells; and *iii*) does not affect apoptosis in hematopoietic cell lines.

## Results

### GA Binding to Scn was Weak and not Affected by Iron

**Figure 2:**
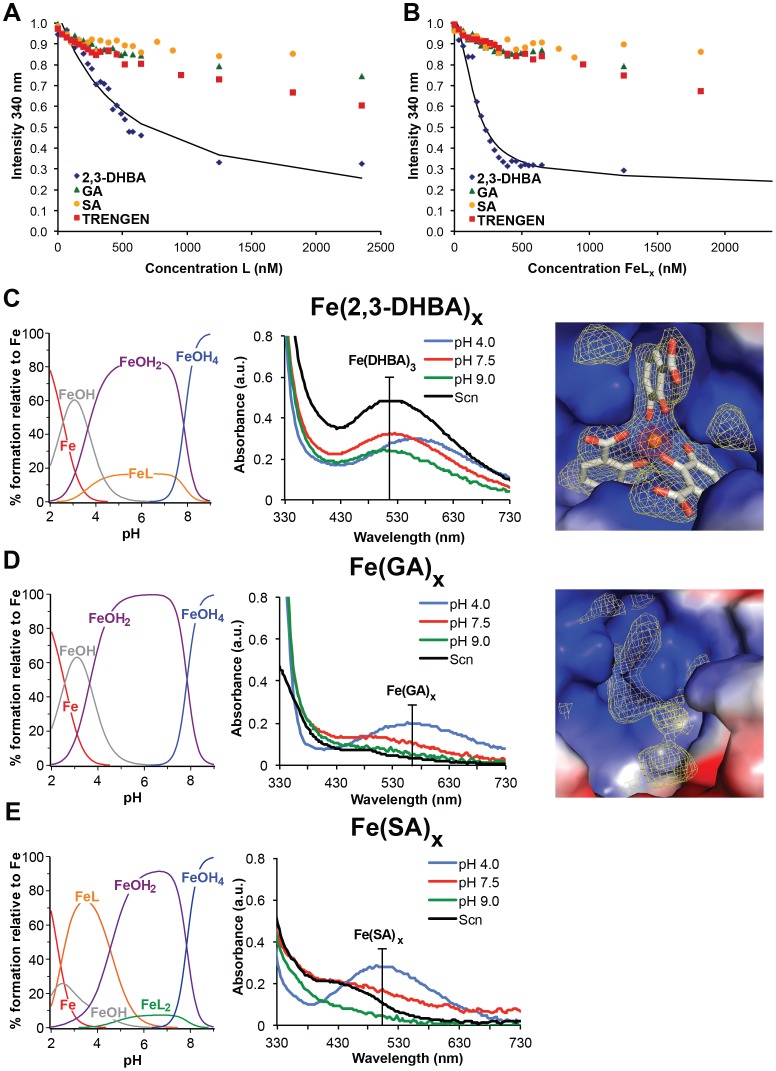
Analysis of the binding of benzoates to iron and Scn. Normalized fluorescence is plotted against concentrations for 2,3-DHBA, SA and GA in the absence (A) or presence (B) of iron. Comparison of the weak quenching by addition of SA, GA or TRENGEN in the presence or absence of iron with 2,3-DHBA responses suggests that SA/Scn, GA/Scn and TRENGEN/Scn dissociation constants, while unfittable by these techniques, would be considerably larger than the derived 2,3-DHBA K_D_ (0.40±0.01 µM). In order to properly model binding in quantitative fluorescence quenching binding assays, solution speciation diagrams (left panels) of iron and 2,3-DHBA (C), GA (D) and SA (E) were calculated with HYSS [17] and confirmed by UV/Vis spectroscopy (middle panels). Right-most panels in (C) and (D) show close-up views of the Scn calyx with Fe(2,3-DHBA)_3_ bound (C) or in the presence of iron/GA mixtures (D) in the same orientation. In these views, the calyx is represented as a molecular surface colored by electrostatic potential; bound ligands are colored by atom-type, with the iron atom shown as an orange sphere. Difference electron density, contoured at 2σ (yellow) and 10σ (red) from delete-refine F_obs_-F_calc_ Fourier syntheses, is shown as nets. Note the absence of any iron peak in (D); residual density in this view can be accounted for by tightly-bound water molecules and the unmodeled side-chain of residue W79, which adopts multiple rotamers.

To qualitatively test binding, an ultrafiltration assay [13] with Ent, catechol, 2,3-DHBA and GA showed greater than 50% iron retention with Ent, catechol and 2,3-DHBA and less than 10% iron retention with GA, comparable to background (Figure S2A). The binding of GA, SA and 2,3-DHBA to human Scn, either without (Figure 2A) or with (Figure 2B) iron, was then analyzed quantitatively at neutral pH using a fluorescence quenching (FQ) assay [13] to compare with the previous FQ analysis of desferri GA binding [16]. The solution speciation as a function of pH was calculated for iron complexation with 2,3-DHBA, GA, SA and catechol ligands (L) under the conditions used in the FQ experiments ([Fe^3+^]  = 20 µM, [L]  = 60 µM; Figures 2C, 2D, 2E and S2B). The quenching of inherent Scn fluorescence upon addition of either desferri or ferric ligands was monitored at the characteristic Scn emission wavelength; *K_D_* values were determined with Hyperquad [17]. Various Fe(2,3-DHBA)_x_ complexes were modeled and those that generated satisfactory fits to the data were based on predominant complex formed at pH 7.2. While both 2,3-DHBA and catechol formed ferric complexes at physiological pH, GA and SA only formed appreciable complexes with iron at low pH. For mixtures of iron and 2,3-DHBA, the Fe:L complex was predominant in solution and was successfully modeled in an association equilibrium with Scn (*K_D_* = 0.101±0.002 nM); the interaction between Scn and desferri 2,3-DHBA was more than a thousand-fold weaker (*K_D_* = 0.40±0.01 µM). Binding models for SA were based on the major solution species, Fe(SA)_2_. While the addition of 2,3-DHBA to Scn resulted in a prominent change in fluorescence, neither the addition of GA or SA, alone or in the presence of iron, showed significant quenching that could be quantitatively analyzed, indicating weak binding to Scn. Since hexadentate *tris*-catecholate siderophores like Ent are more potent iron chelators than their bidentate counterparts, *e. g.* 2,3-DHBA, and may bind more tightly to Scn [3], the *tris*-GA analog TRENGEN was synthesized (Figures 1A and S1). Like GA, TRENGEN did not show significant quenching as desferri or ferric forms, indicating weak binding to Scn (Figures 2A and 2B). The equivalent *tris*-2,3-DHBA analog, TRENCAM, binds to Scn tightly (*K_D_* = 0.32±0.01 nM) [8].

Since Fe(catechol)_x_ complexes were readily observed binding in the Scn calyx by crystallography [13], Scn was co-crystallized in the presence of Fe(2,3-DHBA)_3_ and a 1∶3 mixture of iron and GA to mimic the 2,3-DHBA co-crystallization conditions (Table S1). Initial phases were determined by molecular replacement with a previous Scn structure as the search model (PDB accession code 1L6M). The Scn/Fe(2,3-DHBA)_3_ structure showed clear electron density for three 2,3-DHBA groups and bound iron in the calyx (Figure 2C), while the Scn/Fe/GA structure showed only weak electron density features in the calyx consistent with water molecules and the unresolved side-chain of W79 (Figure 2D), despite ≥millimolar concentrations of protein and GA in the crystallization mix. While the former structure was fully refined (final R_work_/R_free_  = 25.1%/28.8%) and deposited (PDB accession code 3U0D), no further refinement was performed on the empty Scn/Fe/GA structure.

### Exogenous Scn did not Affect Iron Efflux from BOCT-expressing HeLa Cells

**Figure 3:**
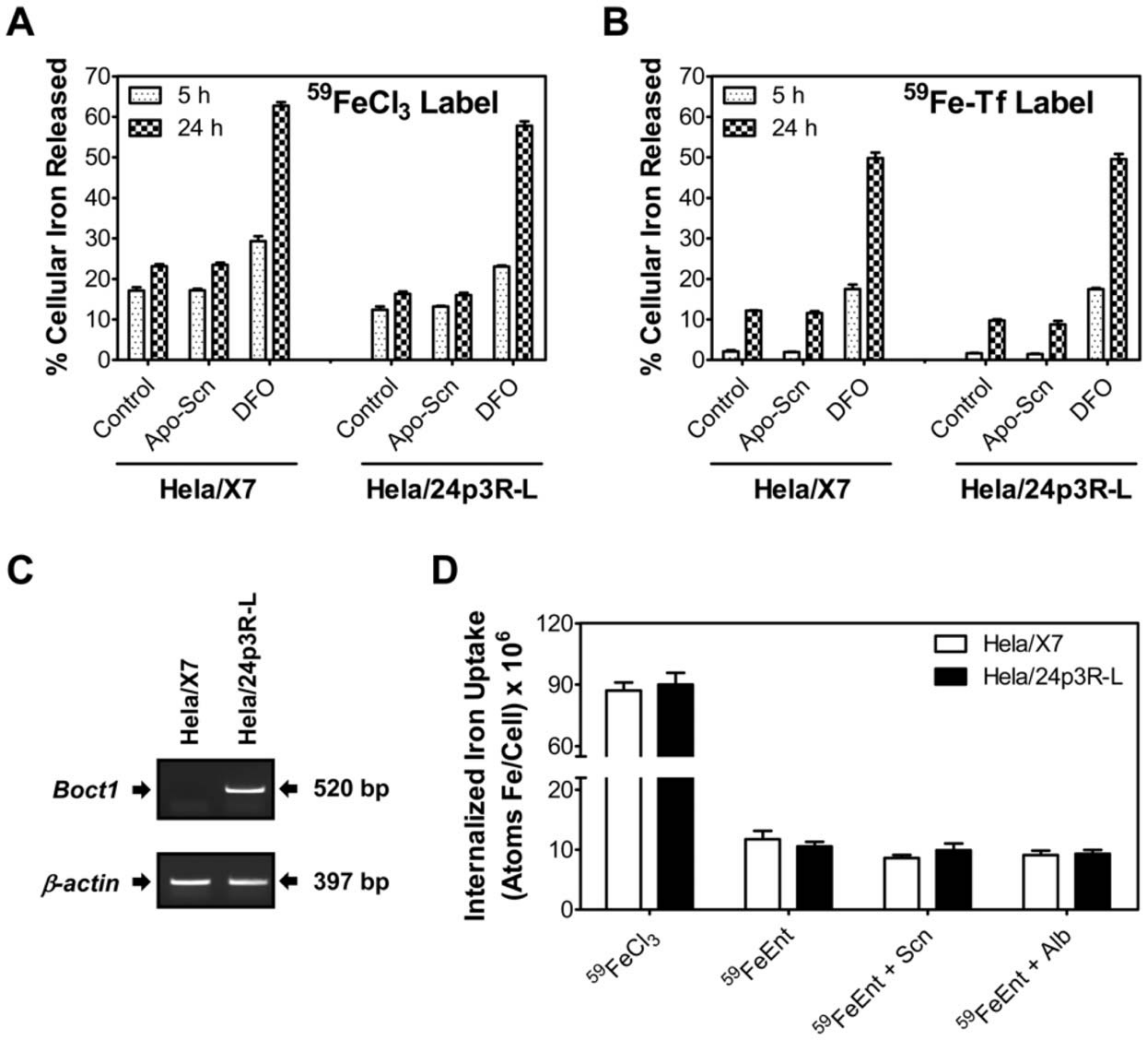
Scn has no effect on iron release or iron uptake from HeLa cells. Control HeLa/X7 (transfected with empty vector) or HeLa/24p3R-L cells were labeled with either (A) 2.5 µM ^59^FeCl_3_ or (B) 0.75 µM ^59^FeTf and re-incubated with 2 µM murine Scn or control medium for 5 h (dotted columns) or 24 h (checked columns); 100 µM DFO was used as a positive control. Expression of *BOCT* in transfected HeLa/24p3R-L cells was confirmed by RT-PCR (C). In (C), a typical result from three experiments is shown. In (D), control HeLa/X7 (white columns) and HeLa/24p3R-L cells (black columns) were incubated for 4 h in the presence of 2 µM ^59^FeCl_3_, 2 µM ^59^FeEnt, 2 µM murine Scn with bound ^59^FeEnt (^59^FeEnt+Scn) or in the presence of 2 µM ^59^FeEnt plus 2 µM human albumin (^59^FeEnt+Alb). Internalized ^59^Fe was determined by γ-counting. Albumin was added in (D) as an additional control for non-specific binding. Error was calculated as the standard deviation among three experiments.

Apo-Scn was reported to markedly increase ^59^Fe release from human HeLa cells transfected with the putative murine Scn receptor, *BOCT* (HeLa/24p3R-L), while apo-Scn had no effect on cells transfected with an empty vector (HeLa/X7) [15]. HeLa/24p3R-L and HeLa/X7 cells obtained from the original investigators were incubated with 2.5 µM ^59^FeCl_3_ for 3 h at 37°C to label intracellular iron pools, washed and then re-incubated for 5 or 24 h at 37°C in the presence or absence of 2 µM apo-Scn. DFO (100 µM) was used as a positive control to mobilize iron from cells [15], [18]. Despite using an identical protocol [15], we did not observe any increase of ^59^Fe release after incubation of HeLa/24p3R-L cells for 24 h with apo-Scn (16.0±0.6%) relative to cells re-incubated with control medium (16.3±0.6%; Figure 3A). After a re-incubation of prelabeled cells for 5 h, less ^59^Fe efflux occurred, but again there was no significant difference in ^59^Fe efflux from HeLa/24p3R-L cells in the presence or absence of apo-Scn (Figure 3A). Apo-Scn also had no effect on increasing ^59^Fe release from control HeLa/X7 cells, while a 24 h re-incubation with DFO markedly and significantly (*p*<0.001) increased ^59^Fe release to 2.7-fold of that found for control medium in HeLa/X7 cells and to 3.5-fold in HeLa/24p3R-L cells (Figure 3A). A 5 h re-incubation of cells with DFO also increased ^59^Fe release relative to control medium alone, although the extent of release was less than that after 24 h, due to the limited permeability of DFO [19]. The ^59^FeCl_3_ concentration used in these experiments (2.5 µM) was 20-fold lower than that used previously [15] to minimize cytotoxicity and non-specific binding of ^59^Fe to the membrane.

Since FeCl_3_ is not a physiologically relevant form of iron, as virtually all iron in the blood of mammals is bound to transferrin (Tf), the studies above were repeated using ^59^FeTf at a concentration ([Tf] = 0.75 µM; [Fe]  = 1.5 µM) within the physiological range found in extracellular fluid [20]. Cells were labeled with ^59^FeTf for 3 h at 37°C and then re-incubated with apo-Scn or DFO for 5 or 24 h at 37°C (Figure 3B), as above. As with ^59^FeCl_3_, a 5 or 24 h re-incubation with apo-Scn did not induce any significant increase in ^59^Fe mobilization from either cell-type. In contrast, after either a 5 or 24 h re-incubation, addition of DFO led to ^59^Fe release from both cell-types. After a 24 h re-incubation with DFO, we observed a significant (*p*<0.001) 4.1-fold (HeLa/X7) and 5.1-fold (HeLa/24p3R-L) increase in ^59^Fe release relative to cells re-incubated with control medium alone. RT-PCR experiments using a primer specifically designed for murine BOCT confirmed that HeLa/24p3R-L cells expressed murine BOCT mRNA, while Hela/X7 cells did not (Figure 3C).

### Exogenous Scn did not Affect Iron uptake by BOCT-expressing HeLa Cells

In contrast to the effect of apo-Scn on iron efflux, holo-Scn was described to be capable of delivering iron to cells [15]. Hence, we tested ^59^Fe uptake from Scn labeled with ^59^FeEnt in HeLa/24p3R-L and HeLa/X7 cells (Figure 3D). Cells were incubated for 4 h at 37°C in serum-free medium with 2 µM ^59^Fe as ^59^FeCl_3_, 2 µM ^59^FeEnt or 2 µM ^59^FeEnt/Scn. Both cell-types internalized similar levels of ^59^Fe from FeCl_3_, although this was 6-to 7-fold greater than ^59^Fe uptake from the ^59^FeEnt in both cell-types. There was no significant difference in the uptake of ^59^FeEnt between HeLa/24p3R-L and HeLa/X7 cells. The greater uptake of ^59^Fe from ^59^FeCl_3_ than ^59^FeEnt by cells can be attributed to the presence of specific transporters on HeLa cells that are known to transport low M_r_ iron [21], [22]. In contrast, the ^59^FeEnt complex did not appear to be transported into cells as effectively as ^59^FeCl_3_, which may be attributable to the larger size and charge of FeEnt [3], [23]. The addition of ^59^FeEnt/Scn to HeLa/24p3R-L and HeLa/X7 cells did not lead to significantly greater uptake than that found for ^59^FeEnt or for ^59^FeEnt mixed with the non-specific control protein albumin. Therefore, Scn did not act to enhance the transport of ^59^FeEnt into HeLa cells in the presence or absence of exogenous BOCT expression.

### Exogenous Scn did not Affect Expression of Iron-responsive Genes

**Figure 4:**
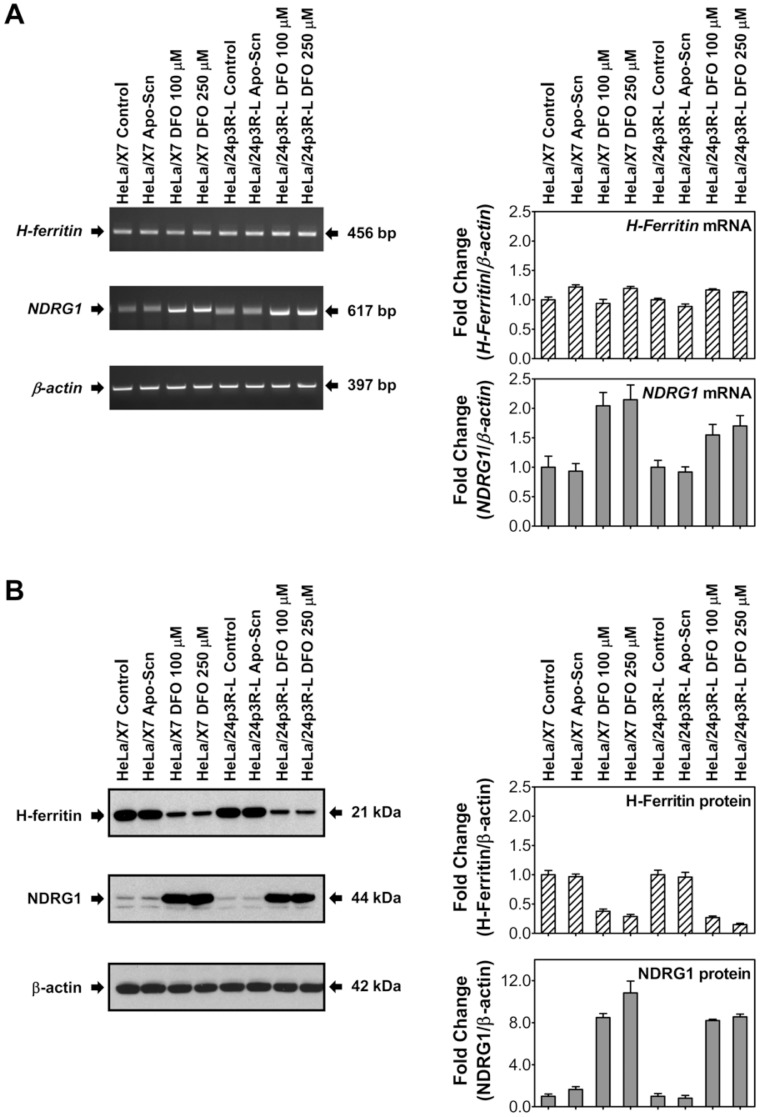
Added Scn does not affect the expression of iron responsive genes. Expression of *H-ferritin* (*FTH-1*) and *NDRG1* in HeLa/X7 and HeLa/24p3R-L cells was assayed by RT-PCR (A) and Western blot (B). Cells were untreated or treated with 2 µM murine Scn or DFO (100 µM or 250 µM) for 24 h. Densitometry results (right) were calculated relative to β-actin; error was calculated from the standard deviation among three experiments; a typical result from three experiments is shown in (A) and (B).

To further assess the effect of apo-Scn on cellular Fe mobilization, the effect of Scn on genes that are sensitively regulated by intracellular Fe levels, *H-ferritin* (heavy polypeptide 1; *FTH1*) and *N-myc downstream regulated gene-1* (*NDRG1*), was monitored (Figure 4). HeLa/24p3R-L and HeLa/X7 cells were incubated for 24 h in control media alone, with 2 µM apo-Scn, or 100 µM or 250 µM DFO. DFO has been shown to up-regulate *NDRG1* mRNA and protein expression [24], [25]. Two bands for NDRG1 were observed, likely representing different phosphorylation states [26], [27]. While addition of DFO markedly up-regulated *NDRG1* mRNA and protein expression, apo-Scn failed to increase *NDRG1* expression (Figure 4). None of the treatments had any significant effect on *H-ferritin* mRNA levels since H-ferritin is regulated by iron at the post-transcriptional level [28]. However, H-ferritin protein expression was decreased by addition of DFO, consistent with previous studies [28], whereas addition of apo-Scn did not have any effect (Figure 4).

### Isolatable BOCT Subdomains do not Bind Scn

**Figure 5:**
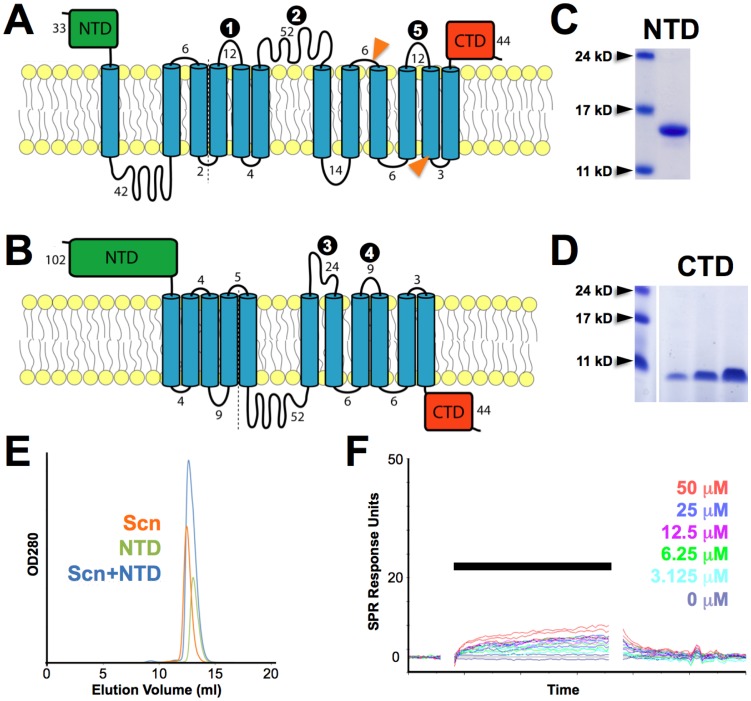
BOCT N-and C-terminal domains do not bind Scn. Predicted BOCT membrane topologies are shown, either as determined in [15] (A) or calculated here (B), with transmembrane-spanning helices shown as blue cylinders. The sequence lengths of the NTD (green), CTD (red) and connecting loops are indicated; loops synthesized as peptides for binding analyses are indicated with numbered black circles, corresponding to the numbering in the **Materials & Methods** section. The amino termini of fragments used to originally identify BOCT as a Scn receptor [15] are indicated with orange arrows in (A). PAGE analyses of bacterially-expressed soluble, purified NTD (C) and CTD (D) are shown. SEC analysis of NTD/Scn is shown in (E). Complex formation would have been indicated by a shift in the Scn+NTD peak to lower elution volumes; in this case, the Scn/NTD mixture runs as the simple summation of the Scn and NTD alone peaks, indicating no binding under these conditions. (F) SPR analysis of Scn/CTD binding, with Scn analyte concentrations indicated. The bar indicates the sample injection period (association phase); gaps in the sensorgrams cover transients associated with injections.

In order to attempt to confirm a functionally-relevant interaction between Scn and its putative receptor BOCT, fragments of BOCT constituting likely independently folded domains or loops predicted to be on the cell surface by previous ([15], Figure 5A) or our own (Figure 5B) topology analyses were synthesized as peptides or recombinantly expressed and purified (Figures 5C and 5D). Since BOCT is a multipass integral membrane protein, the intact receptor is difficult to use in quantitative binding assays; however, multipass receptors often contain identifiable minimal-binding domains that are necessary and sufficient for interactions with ligands. Also, Scn-interacting fragments of BOCT had been identified in prior studies, including a minimal fragment spanning the last predicted transmembrane domain plus the C-terminal 44-residue domain (CTD; Figure 5A) [15], strongly suggesting that the CTD would be sufficient to mediate Scn binding. However, none of these peptides or domains, including a soluble form of the CTD, displayed measurable affinities for Scn by size exclusion chromatography (SEC; an example result is shown in Figure 5E) or surface plasmon resonance (SPR; an example result is shown in Figure 5F). Additional binding assays using isothermal titration microcalorimetry or co-crystallization also failed to show measurable interactions (*data not shown*).

### Exogenous Scn does not Drive Apoptosis in Murine Hematopoietic Cell Lines

**Figure 6:**
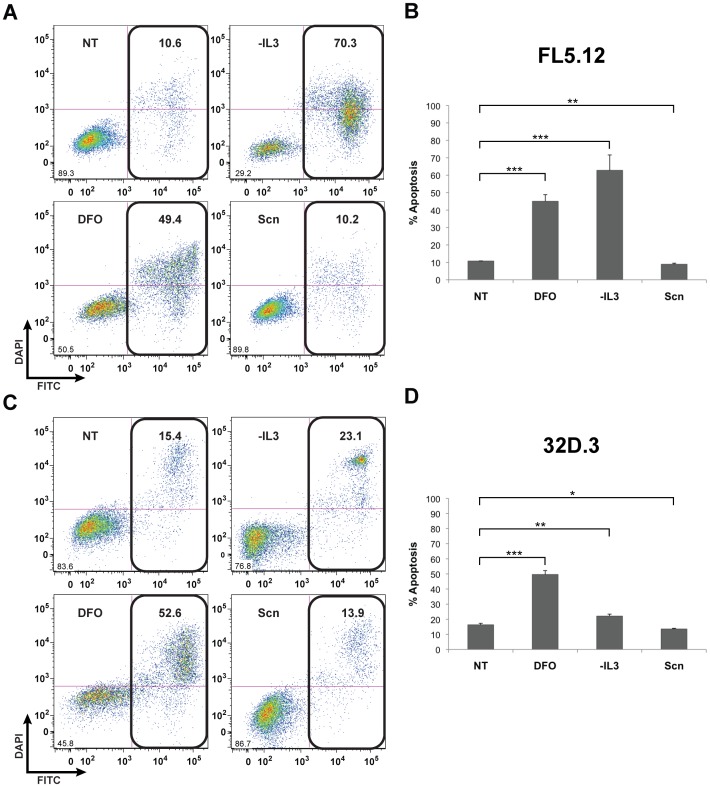
Scn does induce apoptosis in murine 32D.3 or FL5.12 cells. FL5.12 (A) and 32D.3 (C) cells were incubated with 10 µM Scn and DFO for 48 h (NT: no treatment; -IL-3: in the absence of added IL-3). Apoptosis was assayed by annexin V-FITC staining and DAPI was used as a vital stain; percentages of cells positive for annexin staining are indicated. Average annexin V-positivity from three independent experiments are shown for FL5.12 (B) and 32D.3 (D) cells; error was calculated as the standard deviation of three replicates. Statistical significance is indicated as *p<0.05; **p<0.01; ***p<0.001. Note that while the effect of adding Scn was significant, the effect was *anti*-apoptotic.

It had been reported that 32D.3 or FL5.12 cells undergo apoptosis upon addition of apo-Scn at concentrations up to 0.5 µM [14]. However, while IL-3 withdrawal or addition of 10 or 100 µM DFO induced robust apoptotic responses in 32D.3 and FL5.12 cells after 48 h, recombinant apo-Scn, added at concentrations of 10 or 100 µM (20-or 200-fold higher concentrations than used previously), did not induce apoptosis in 32D.3 or FL5.12 cells (Figures 6 and S3). Indeed, addition of recombinant Scn at these concentrations had a significant (*p*<0.05) *anti*-apoptotic effect (Figure 6). Scn used in these and prior experiments was expressed recombinantly in *E. coli*, but Scn expressed in HEK293-F cells [29], retaining native glycosylation, yielded comparable results (*data not shown*).

### 32D.3 and FL5.12 Cells Stably Transduced with Scn are Viable

**Figure 7:**
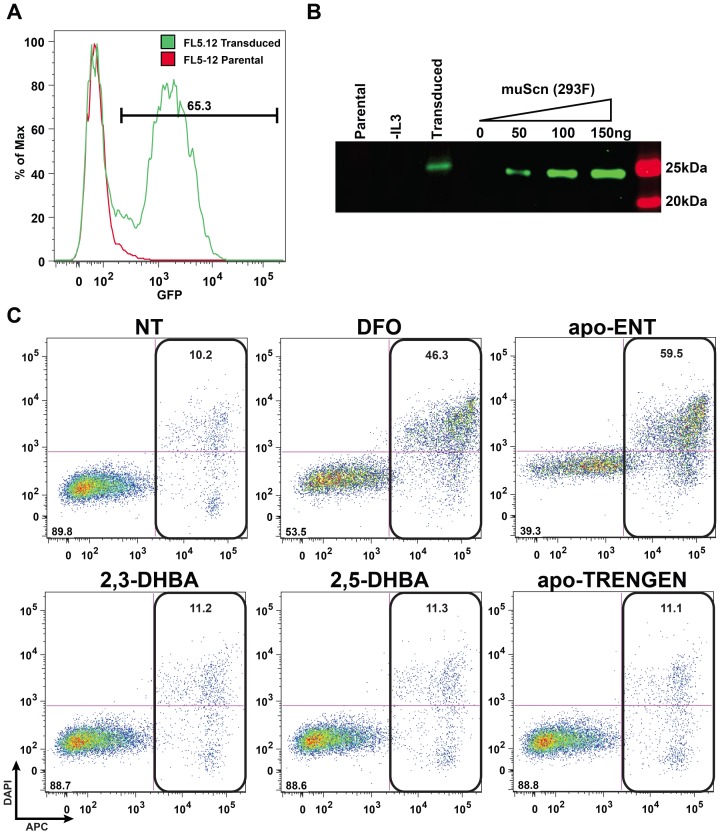
Stably-induced expression of Scn does not drive apoptosis in FL5.12 cells. (A) FL5.12 cells were transduced with the pCVL-SFFV-muScn-IRES-GFP lentivirus and GFP mean fluorescence intensity was determined one-week post-transduction by cytometry, confirming transgene functionality. (B) A Western blot of supernatants, concentrated from 32 µL, from FL5.12 cells shows that the transduced cells constitutively express Scn, while parental cells in the presence or absence of IL-3 do not secrete detectable amounts of Scn after 72 h in culture. (C) Transduced FL5.12 were incubated with a variety of siderophores in order to assess the role of exogenous siderophores on cell viability (NT: no treatment). The hexadentate chelators DFO and Ent at 100 µM produce robust apoptosis, while the bidentate chelators at 300 µM do not affect viability.

To mimic the proposed autocrine mechanism of Scn-mediated apoptosis [14], 32D.3 and FL5.12 cells were induced to stably secrete murine Scn at ∼2 mg/L levels with a lentivirus construct [29] (Figures 7A, 7B and S4A). These cells show normal levels of viability in the presence of IL-3, but undergo apoptosis as expected in response to the addition of DFO (Figures 7C and S4B), which induces cellular iron-depletion [30]. In order to eliminate the possibility that the levels of endogenous siderophore available *in vitro* were limiting for a hypothetical autocrine effect of Scn on apoptosis under these conditions, iron-free Ent, 2,3-DHBA, 2,5-DHBA and TRENGEN were added at 100 µM concentrations (Figures 7C and S4B). Addition of Ent at this concentration induced robust apoptosis in transduced 32D.3 and FL5.12 cells while none of the other compounds had significant effects on viability, together showing that Scn does not induce apoptosis through an autocrine mechanism and supporting the hypothesis that bidentate siderophores and TRENGEN do not chelate iron strongly enough to affect iron metabolism *in vitro*.

### 32D.3 and FL5.12 Cells do not Secrete Detectable Scn in Response to IL-3 Withdrawal

The initial observation underlying the Scn-apoptosis hypothesis *via* iron-depletion was the up-regulation of Scn in response to cytokine withdrawal [14]. As a control for the levels of Scn secreted from transduced cells, the levels of Scn secreted from 32D.3 and FL5.12 cells undergoing IL-3 withdrawal-induced apoptosis were measured by Western analyses (Figures 7B and S4A). However, no detectable Scn was observed in concentrated supernatants from 32D.3 and FL5.12 cells undergoing apoptosis.

### An Anti-Scn Antibody does not Block Apoptosis

**Figure 8:**
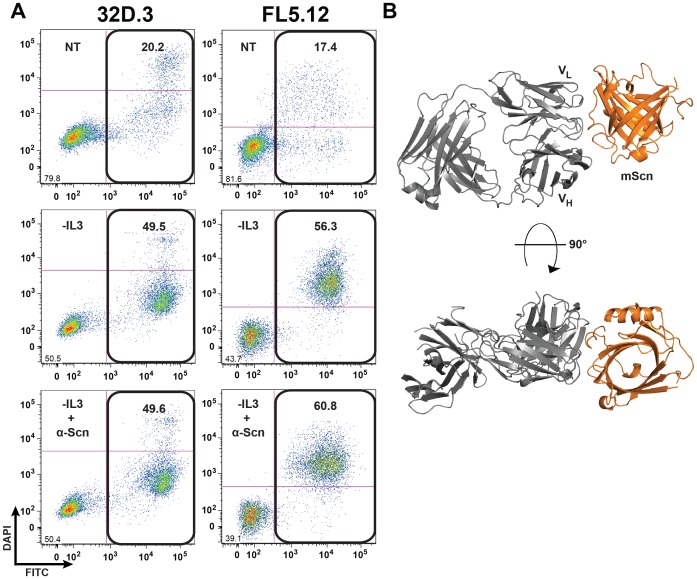
An anti-Scn antibody does not block IL-3 withdrawal-induced apoptosis. (A) 32D.3 and FL5.12 cells in the presence (NT) or absence (-IL3) of IL-3 were incubated for 48 h with 10 µM of the anti-Scn antibody MAB1857; percent annexin-V positivity is indicated. (B) The structure of Fab MAB1857 with Scn, shown in a ribbon representation (Fab in gray and Scn in orange), reveals the interface that is occluded in the complex. Had Scn had an effect on apoptosis through receptor-mediated uptake, the effect of the antibody on the process would have identified a potential receptor-interacting surface on Scn, the rationale for this approach. However, since Scn does not affect apoptosis, an anti-Scn antibody cannot reveal a receptor-interacting surface by blocking a non-existent effect.

In order to begin mapping interactions between Scn and cell-surface receptors mediating endocytosis and subsequent apoptosis, an anti-Scn antibody (R&D Systems MAB1857) was tested for the ability to affect IL-3 withdrawal-induced apoptosis in 32D.3 and FL5.12 cells (Figure 8A) and co-crystallized with murine Scn as an Fab fragment to determine its binding footprint on Scn (Figure 8B and Table S1). However, since Scn did not drive apoptosis in these cells in the above experiments, this analysis was not informative, though characterizing this interaction is useful for future studies of interactions between Scn and bona fide receptors.

## Discussion

The proposal that GA, or siderophores that incorporate GA substituents, would bind tightly to Scn contradicted a series of studies detailing the recognition mechanism and specificity of Scn [2]–[5], [7]–[9], [13], [31], [32]. Modifications to the catechol functional group that widen the complex with iron, such as 3,4-DHBA (protocatechuic acid) substituents in the anthrax siderophore petrobactin [9], glucose modifications of the salmochelins [4] or adducts in synthetic siderophore analogs [9], do not bind to Scn as iron complexes because of steric clashes with the Scn calyx. At neutral pH, Ent and 2,3-DHBA chelate ferric ions in solution through the adjacent catechol 2-and 3-OH groups, generating complete, hexadentate FeEnt complexes or incomplete Fe(2,3-DHBA)_x_ complexes. As the pH is lowered, iron binding shifts from catecholate-mode to salicylate-mode, engaging the 2-OH and carbonyl oxygens, and then to iron release [33], [34]. The shift from catecholate-to salicylate-mode binding is accompanied by an outward swing of the catechol groups, widening the ferric complex to a diameter that is also sterically incompatible with two of three pockets in the rigid calyx of Scn. Ferric complexes of SA and obligate salicylate-mode analogs of Ent [7] do not bind to Scn for this reason.

This transition is illustrated in the Fe(2,3-DHBA)_3_ structure reported here (Figure 2C); crystallized at low pH, one of the three 2,3-DHBA moieties shifted to salicylate-mode binding in the only pocket where this transition is tolerated. GA, because it lacks neighboring (*ortho*) hydroxyl groups, must chelate iron in salicylate-mode. The position of the 5-OH group of GA maximizes collisions with the Scn calyx, accentuating the steric clash, though the weak binding of ferric SA complexes to Scn showed that salicylate-mode binding is sufficient on its own to ablate binding. Therefore, ferric complexes with GA or siderophores with GA substituents should not bind to Scn with appreciable affinity, confirmed here in qualitative, quantitative and crystallographic binding studies, demonstrating that GA alone cannot facilitate iron binding by Scn. The synthetic *tris*-GA compound TRENGEN also failed to show measurable interactions with Scn in contrast to the analogous synthetic *tris*-2,3-DHBA analog TRENCAM which binds tightly [8].

Scn generally crystallizes at pHs in the 4 to 4.5 range. While this is below the release pH for catechol complexes, a partially-released complex was observed because of the high concentration of components and the very non-physiological conditions in the crystallization trials [13]. Low pH release of iron from Scn complexes is a necessary component of the iron delivery hypothesis. However, the apoptosis through iron withdrawal hypothesis requires tight binding of iron in Scn complexes at both neutral and low pHs, to both efficiently sequester iron intracellularly and outcompete Tf extracellularly, otherwise iron would simply be returned to cells through normal trafficking. However, speciation analyses showed little SA/iron and essentially no GA/iron complex formation at neutral pH, while the binding studies showed no GA/Scn ligation at neutral or low pHs.

The inability to confirm the iron-or Scn-binding properties of GA led to a reconsideration of the remaining elements of the Scn-induces-apoptosis hypothesis laid out in three seminal papers [14]–[16]. Despite using the same experimental models, exogenously-added Scn did not affect iron efflux or uptake, or affect the expression levels of iron-responsive genes, in BOCT-expressing HeLa cells. While failing to confirm the role of Scn in iron mobilization, these results also failed to demonstrate a functional association between Scn and BOCT, consistent with recent results that failed to demonstrate binding between the rat orthologs of Scn and BOCT [35] and our own inability to identify a murine BOCT subdomain sufficient to mediate murine Scn binding. Furthermore, exogenously-added Scn did not drive apoptosis in IL-3 dependent murine hematopoietic cell lines, even at levels 200-fold higher than reported by Devireddy and coworkers to induce robust responses. In fact, stable, Scn-secreting transductants of these cell lines were viable in culture even in the presence of added GA or 2,3-DHBA, but readily apoptose in the presence of added DFO or Ent. Hexadentate chelators, like DFO and Ent, are able to effectively compete with Tf for iron, but bidentate chelators, like 2,3-DHBA, while effective at solubilizing iron in solution, do not display affinities sufficient to outcompete Tf for iron [36]. GA and TRENGEN, which cannot bind iron at the neutral pH used in cell culture studies, did not significantly affect iron metabolism in these experiments. Finally, FL5.12 and 32D.3 cells undergoing apoptosis in response to IL-3 withdrawal did not secrete detectable levels of Scn.

The failure of GA to function as a siderophore under physiological conditions suggested a reexamination of the logic behind the identification of DHRS6, as DHRS6 was not directly shown to catalyze the synthesis of GA from any hypothetical precursor [16]. DHRS6 was identified on the basis of sequence homology to the enterobacterial enzyme *trans*-2,3-dihydro-2,3-dihydroxybenzoate dehydrogenase (EntA), which catalyzes the conversion of 2,3-diDHBA to 2,3-DHBA as part of Ent biosynthesis: DHRS6 is the closest mammalian homolog of EntA [16]. However, the converse is not true; the closest bacterial homologs of DHRS6 are a family of specific hydroxybutyrate dehydrogenases, with structural features associated with hydroxybutyrate binding in DHRS6 (three arginine residues) conserved across vertebrate DHRS6 orthologs and at least one bacterial DHRS6 ortholog (from *Bordetella bronchiseptica*) [37]. *B. bronchiseptica* produces the hydroxamate-type siderophore alcaligin, not catecholate siderophores [38], so does not require an EntA-like activity for iron acquisition, consistent with annotating the *B. bronchiseptica* protein as a hydroxybutyrate dehydrogenase and not as an EntA analog. DHRS6 is a highly specific enzyme, showing considerable activity against (*R*)-OH butyrate, but no measurable activity against the closely related compounds (*S*)-OH butyrate, 3-OH-*R*-2-methylbutyrate or 3-OH-*S*-2-methylbutyrate, consistent with the tight constraints imposed by a highly specific substrate binding site [37]. EntA is also highly selective, efficiently converting 2,3-diDHBA but poorly tolerating substituents on the 4 and 5 positions [39], as on diGA. While EntA is unlikely to efficiently catalyze conversion of both diGA and 2,3-diDHBA substrates, it is possible that a vertebrate EntA analog could, though this should be formally demonstrated since indiscriminate conversion of dihydroxybenzoate isomers would be unusual for this class of enzymes. However, the expectation would be that DHRS6 is simply a highly stereospecific hydroxybutyrate dehydrogenase with insufficient reactivity towards unrelated substrates, like diGA, to generate GA.

The logical framework of the hypothesis that Scn drives apoptosis of hematopoietic cells through iron depletion, mediated by interactions with GA as endogenous siderophore and BOCT as cell-surface receptor, constitutes an interdependent chain predicated on the integrity of each experimental link. We have shown that multiple links in this chain are questionable on the basis of first principles in the absence of direct experimental support (*i.e.,* DHRS6 catalyzes the production of GA) or cannot be reproduced (*i.e.*, GA binds to Scn, GA is a siderophore, BOCT mediates Scn iron export, Scn drives apoptosis, Scn is secreted in response to cytokine withdrawal). In light of these results, where any single break in the logical chain invalidates the overall hypothesis, the endogenous role of Scn in apoptosis needs to be fully reevaluated.

## Materials and Methods

### Filter Retention Binding Assay

Apo-Scn (10 µM), ^55^Fe^III^ (1 µM), cold Fe^III^Cl_3_ (9 µM) and a candidate siderophore (10 µM) were incubated in 150 mM NaCl, 20 mM Tris (pH 7.4) and incubated at ambient temperature for 60 min as described in [13]. The mixture was then washed four times with the Tris buffer on YM-10 ultrafilters (Millipore) and the retained ^55^Fe measured with a scintillation counter.

### FQ Binding Assay

GA was obtained from TCI America (min. 98% purity) and TRENGEN was synthesized as described in Experimental Procedures S1; FeCl_3_ stock solutions in 1 M HCl were standardized by EDTA titration [40]. Quenching of human Scn was measured on a Cary Eclipse fluorescence spectrophotometer (20 nm slit band pass for excitation; 2.5 nm slit band pass for emission) using characteristic Scn excitation and emission wavelengths, 281 nm and 320–340 nm, respectively. Measurements were made at a protein concentration of 100 nM in Tris-buffered saline (TBS; pH 7.2), 5% DMSO, plus 32 µg/mL ubiquitin. Fluorescence intensities were corrected for dilution due to addition of ligand. An aliquot of a DMSO stock solution of the free ligand (12 mM; 25 µL) and FeCl_3_ salt (27 mM, 3.7 µL, 0.33 equivalents) were combined and diluted with TBS (pH 7.2) to form the metal complexes at a concentration of 0.1 mM (no metal added for apo-ligands). The solutions were equilibrated for 1.5 h and diluted to a final concentration of 20 µM in 5% DMSO/TBS buffer. Fluorescence data were analyzed by a non-linear regression analysis (Figures 2A and 2B) of the normalized fluorescence response versus ligand concentration using Hyperquad [17]. The model for determination of the species stoichiometry and *K_D_* took into account ferric ion hydrolysis constants [41], 2,5-DHBA protonation constants [42] and Fe-2,5-DHBA formation constants [42]. Dissociation constants were determined from at least three independent titrations.

### UV/Vis Spectroscopy

Stock solutions (22.5 mM) of catechol, 2,3-DHBA, GA and SA (Sigma-Aldrich) were prepared in ultrapure water; a FeCl_3_ stock was prepared at 500 mM in 1 M HCl. Iron complex solutions were prepared at 4.5 mM siderophore and 1.5 mM FeCl_3_ in 100 mM sodium acetate (pH 4.0), Tris (pH 7.5) or Tris (pH 9.0). Scn (6 mg/mL) in 100 mM Tris (pH 7.5) was mixed with each of the ferric siderophores and extensively washed with Tris (pH 7.5) through multiple rounds of ultrafiltration to remove any unbound ligand. Absorbance data (Figures 2C, 2D and 2E) were collected immediately at ambient temperature using a Nanodrop ND-1000 spectrometer (Thermo Scientific).

### Crystallography

Crystals of Scn, loaded with molar excesses of stoichiometric iron/GA or iron/2,3-DHBA mixtures and subsequently washed and concentrated by ultrafiltration, were grown as previously described [13]. Scn/Fab complexes were prepared by cleaving monoclonal anti-Scn rat IgG_2A_ (R&D Systems MAB1857) with papain, adding Scn and purifying the complex by size exclusion chromatography. Crystals were grown by vapor diffusion at 25°C: protein at 10 mg/mL was mixed 1∶1 with a reservoir solution of 0.1 M sodium citrate (pH 4.2), 0.2 M sodium chloride and 20% w/w PEG 8000. Crystals were cryopreserved in reservoir solution plus 15% v/v glycerol. Diffraction data were collected at the Advanced Light Source, beamline 5.0.1.

### PCR Primers

For murine *BOCT* (GenBank entry NM_021551), the sense primer used was 5′-AAGCGGCAGATTGAGGAA-3′ and anti-sense primer was 5′-CTTCAGAAGCAAGGAGGGTAC-3′. For human *NDRG1*, the sense primer used was 5′-TCACCCAGCACTTTGCCGTCT-3′ and the anti-sense primer was 5′-GCCACAGTCCGCCATCTT-3′. For human *H-ferritin (FTH1*), the sense primer used was 5′-CCTCCTACGTTTACCTGTC-3′ and anti-sense primer was 5′-TTTCATTATCACTGTCTCCC-3′. For human *β-actin*, the sense primer used was 5′-CCCGCCGCCAGCTCACCATGG-3′ and the anti-sense primer was 5′-AAGGTCTCAAACATGATCTGGGTC-3′.

### HeLa Iron Transport Assays

Human apo-Tf (Sigma-Aldrich) was labeled with ^59^Fe (PerkinElmer) to produce diferric ^59^FeTf using the ferric nitriloacetate complex at a iron:nitriloacetate molar ratio of 1∶10 as previously described [43]. The iron saturation of Tf was monitored by UV-Vis spectrophotometry comparing the absorbance at 280 nm (protein) with that at 465 nm (iron-bound complex). HeLa/24p3R-L and HeLa/X7 cells [15] were kindly provided by M. R. Green (University of Massachusetts Medical School). 24p3R-L refers to a widely expressed, longer splice variant of BOCT as compared to a short splice variant lacking the N-terminal 154 amino acids [15]. Cells were cultured as described [15] using DMEM (Invitrogen) supplemented with 10% fetal calf serum (Invitrogen) and 2.5 µg/mL blasticidin (Sigma-Aldrich). To confirm expression of *BOCT*, total RNA was isolated using TRIzol® (Invitrogen) and RT-PCR was performed using SuperScript III RT/Platinum® Taq Mix as previously described [44] using primers detailed as above. Western blot analysis was performed using established protocols [45] and primary antibodies against NDRG1 (Abcam 37897), H-ferritin (Cell Signaling Technology 3998) and β-actin (Sigma-Aldrich A5441). For ^59^Fe release experiments (Figure 3), cells growing as a monolayer were pre-labeled with 0.75 µM ^59^FeTf for 3 h at 37°C in DMEM (Invitrogen) plus 10% fetal calf serum (Invitrogen). Cultures were then washed four times with PBS on ice and then re-incubated in fresh culture media with or without 2 µM apo-Scn for 5 or 24 h at 37°C; 100 µM DFO (Novartis or Sigma-Aldrich) was used as positive control. Scn was obtained from R&D Systems, the kind gift of L. Devireddy (Case Western Reserve University) or was produced as previously described [46]. After this incubation, the supernatant was collected and the cells harvested to estimate radioactivity using a 2480 Wizard^2^ γ-counter (PerkinElmer). In additional experiments, cells were pre-labeled with 2.5 µM ^59^FeCl_3_ (PerkinElmer) instead of ^59^FeTf as in [15]. For ^59^Fe uptake experiments (Figure 3), ^59^FeEnt was produced by incubating iron-free Ent (EMC Microcollections) with ^59^FeCl_3_ (PerkinElmer) in a molar ratio of 1∶1 for 30 min at 37°C in the dark. Scn was incubated with ^59^FeEnt in a 1∶1 molar ratio at 37°C for 30 min in the dark to generate radiolabeled holo-Scn, albumin was similarly pre-incubated with ^59^FeEnt. To measure iron uptake, cells were incubated in serum-free DMEM (Invitrogen) with 2 µM ^59^FeCl_3_, 2 µM ^59^FeEnt or 2 µM holo-Scn for 4 h at 37°C. Human albumin (2 µM) was added with ^59^FeEnt as a control for non-specific protein-binding and transport. After this incubation, cells were washed on ice four times with PBS and harvested for γ-counting. Experiments were performed in triplicate and data were compared using Student’s *t*-test; results were considered statistically significant when *p*<0.05.

### Expression and Characterization of BOCT Subdomains

Predicted membrane topologies of BOCT, determined by Devireddy and coworkers (Figure 5A) [15] or the union of results from several computational algorithms (TMHMM [47], [48], TMpred [49], SOSUI [50]), suggest that N-and C-terminal BOCT sequences both comprise domains large enough to form independent folding units (NTD: in its longer form, residues 1 through 102; CTD: residues 477 through 520). NTD^1–102^ and CTD^477–520^ were expressed recombinantly in *E. coli*, the former as a His-tagged, periplasmically-targeted construct and the latter as a cytoplasmically-targeted, cleavable GST-fusion (Figures 5C and 5D). Binding of NTD or CTD to Scn was assayed by SEC (*e. g.*, Figure 5E) and SPR (*e. g.*, Figure 5D). In Figure 5D, 2086 SPR response units (RUs) of Scn were amine-coupled to CM5 sensor chips (Biacore) following the manufacturer’s protocol. CTD analytes, at concentrations from 3.125 to 50 µM, were injected in duplicate, in random order, for one minute at a flow rate of 20 µl/min on a Biacore 3000 system. Sensorgrams were blank-corrected by the double-subtraction method [51], using a capped channel as blank. In this experiment, a saturating response on a fully-active surface would correspond to >100 RUs; therefore, the very weak responses observed, even at very high analyte concentrations, show that the CTD/Scn interaction has an equilibrium dissociation constant considerably weaker than 50 µM. Comparable results were obtained for NTD. Peptides corresponding to predicted cell-surface loops of significant length (>6 residues) in either topology were synthesized commercially (Genscript) with N-terminal biotin groups:

1. b-SKDWRFLQR (residues 210 through 218)
2. b-ESARWLIVKRQIEEAQSVLRILAERNRPHGQMLGEEAQEALQELENTSPLPATSTFS (residues 238 through 294)
3. b-FTNFIAHAIRHSYQPVGGGGSPSD (residues 313 through 336)
4. b-WDYLNDAAITT (residues 386 through 396)
5. b-QRLHMGHGAFLQ (residues 446 through 457)

BOCT loop peptides were coupled to strepavidin-coated sensor chips and analyzed with Scn analytes using analogous methodology; as above, no quantifiable responses were detected (*data not shown*).

### Apoptosis Assays

32D.3 (ATCC CRL-11346) and FL5.12 (the kind gift of L. Devireddy (Case Western Reserve University)) cells were cultured in modified RPMI-1640 (ATCC) containing 5% fetal calf serum and 5 ng/mL murine IL-3 (BD). FL5.12 and 32D.3 cells were maintained in culture at 5×10^5^ cells/mL and 24 h later seeded at 1×10^5^ cells/mL in 24 well plates. 10 µM DFO or Scn was added to the cells and incubated for 48 h; Scn was produced as previously described [46]. Annexin V-FITC/DAPI staining was carried out as described by the manufacturer (BD) and each sample was analyzed by flow cytometry. Transduction of 32D.3 and FL5.12 cells was carried out at 1×10^6^ cells/mL in media supplemented with 4 µg/mL hexadimethrine bromide. The lentiviral construct used for the transductions was described previously [29]. Experiments were performed in triplicate and data were compared using Student’s *t*-test; results were considered statistically significant when *p*<0.05.

## Supporting Information

Figure S1 **Related to** **Figure 1:** **Synthesis of TRENGEN.**
(TIF)

Figure S2 **Related to** **Figure 2:** **Catechol solubilizes iron at neutral pH and mediates iron retention by Scn.** (A) Iron retention by Scn in an ultrafiltration assay in the presence of various candidate siderophores is shown; error was calculated from the standard deviation of triplicate experiments. (B) HYSS speciation analysis (left panel) and UV/Vis spectroscopic analysis of iron/catechol/Scn interactions.
(TIF)

Figure S3 **Related to** **Figure 6:** **Scn does not induce apoptosis at high concentrations.** FL5.12 (A) and 32D.3 (C) cells were incubated with 100 µM Scn and DFO for 48 h. Apoptosis was assayed by annexin V-FITC staining and DAPI was used as a vital stain; percentages of cells positive for annexin staining are indicated.
(TIF)

Figure S4 **Related to** **Figure 7:** **Stably-induced expression of Scn does not drive apoptosis in 32D.3 cells.** (A) A Western blot of 32D.3 cells shows that the transduced cells constitutively express Scn, while parental cells in the presence or absence of IL-3 do not secrete detectable amounts of Scn after 72 h in culture; 32 µL of culture supernatants was concentrated and loaded in the first three lanes. (B) Transduced 32D.3 were incubated with a variety of siderophores in order to assess the role of exogenous siderophores on cell viability. The hexadentate chelators DFO and Ent at 100 µM produce robust apoptosis, while the bidentate chelators at 300 µM do not affect viability.
(TIF)

Table S1 **Related to** **Figure 8:** **Crystallographic statistics.**
(DOCX)

Experimental Procedures S1 **TRENGEN synthesis.**
(DOCX)
