# Supplementary figures and images for: Siderocalin/Lcn2/NGAL/24p3 Does Not Drive Apoptosis Through Gentisic Acid Mediated Iron Withdrawal in Hematopoietic Cell Lines
Source: PLoS One. 2012 Aug 21;7(8):e43696. doi: 10.1371/journal.pone.0043696 (PMC3424236; doi:10.1371/journal.pone.0043696)

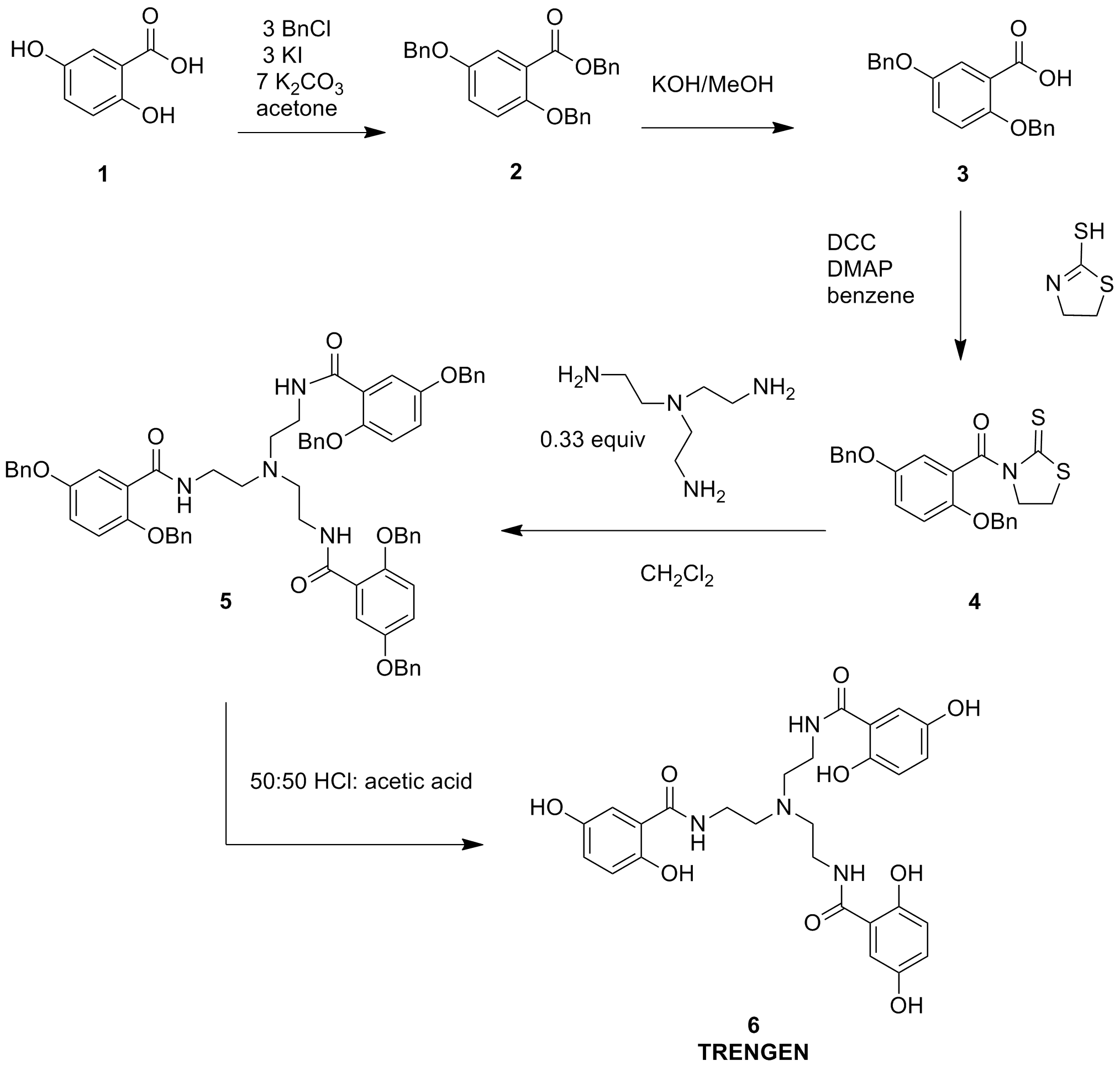

Supplement: Figure S1 — Related to Figure 1: Synthesis of TRENGEN. (TIF) [file pone.0043696.s001.tif]

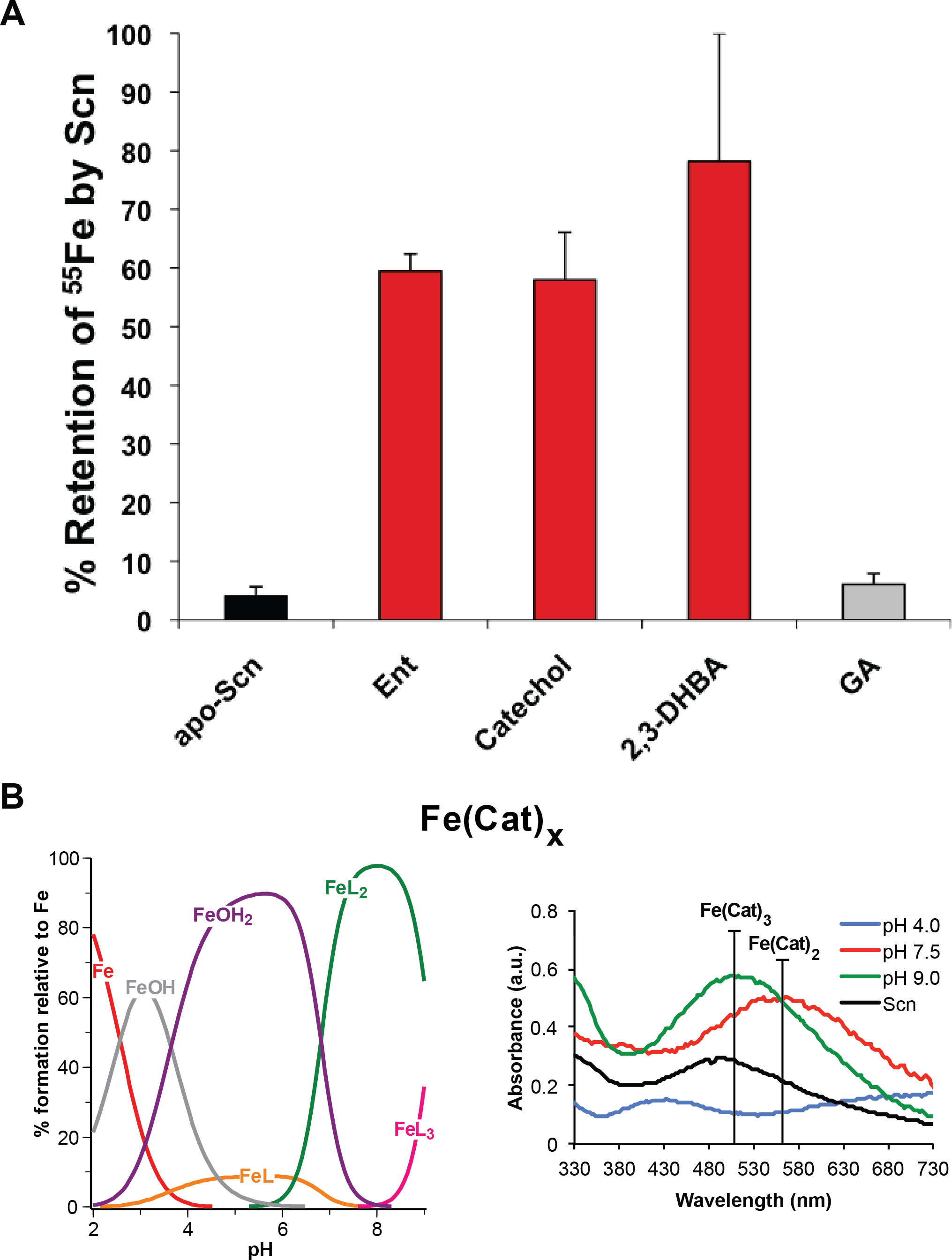

Supplement: Figure S2 — Related to Figure 2: Catechol solubilizes iron at neutral pH and mediates iron retention by Scn. (A) Iron retention by Scn in an ultrafiltration assay in the presence of various candidate siderophores is shown; error was calculated from the standard deviation of triplicate experiments. (B) HYSS speciation analysis (left panel) and UV/Vis spectroscopic analysis of iron/catechol/Scn interactions. (TIF) [file pone.0043696.s002.tif]

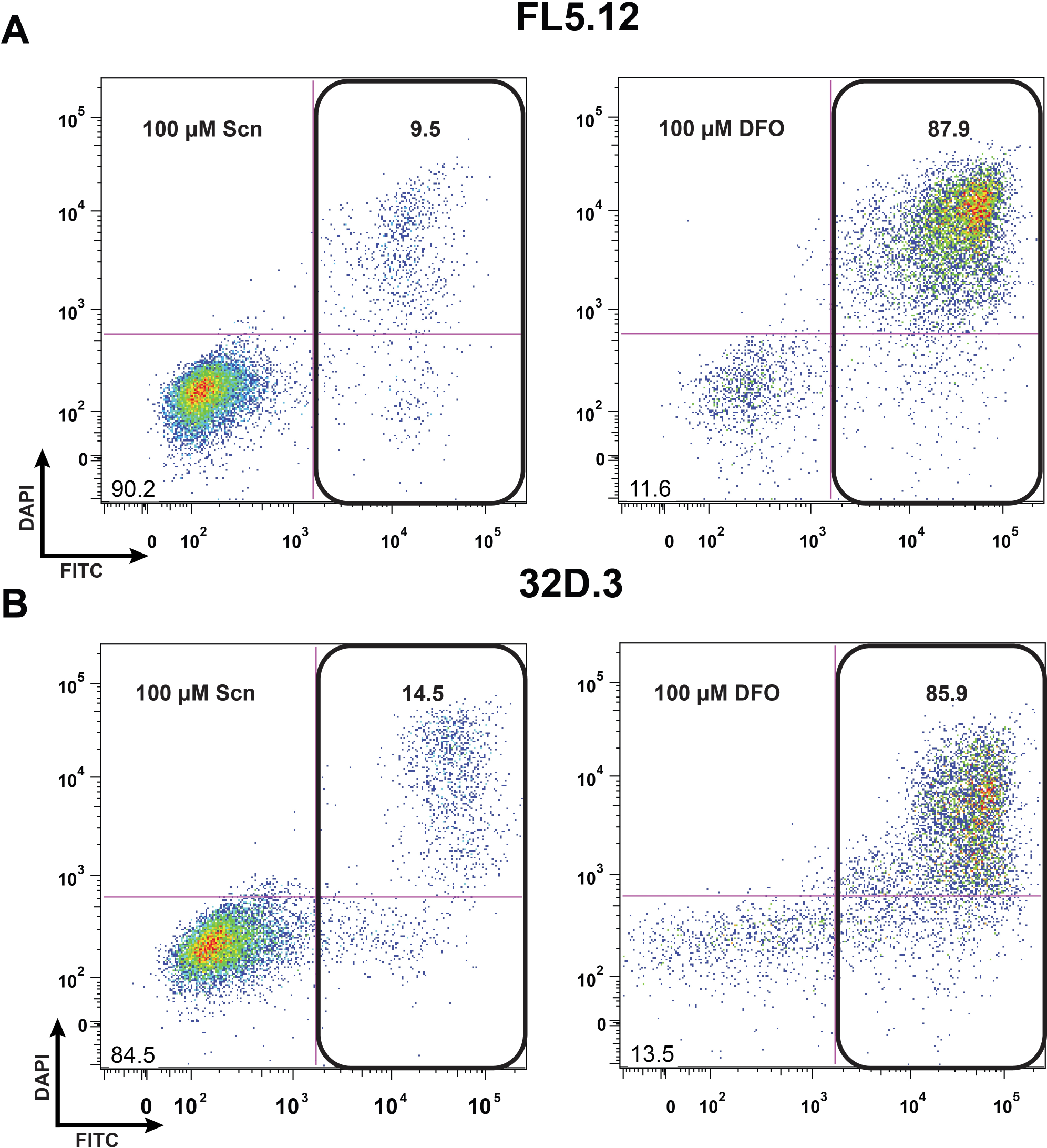

Supplement: Figure S3 — Related to Figure 6: Scn does not induce apoptosis at high concentrations. FL5.12 (A) and 32D.3 (C) cells were incubated with 100 µM Scn and DFO for 48 h. Apoptosis was assayed by annexin V-FITC staining and DAPI was used as a vital stain; percentages of cells positive for annexin staining are indicated. (TIF) [file pone.0043696.s003.tif]

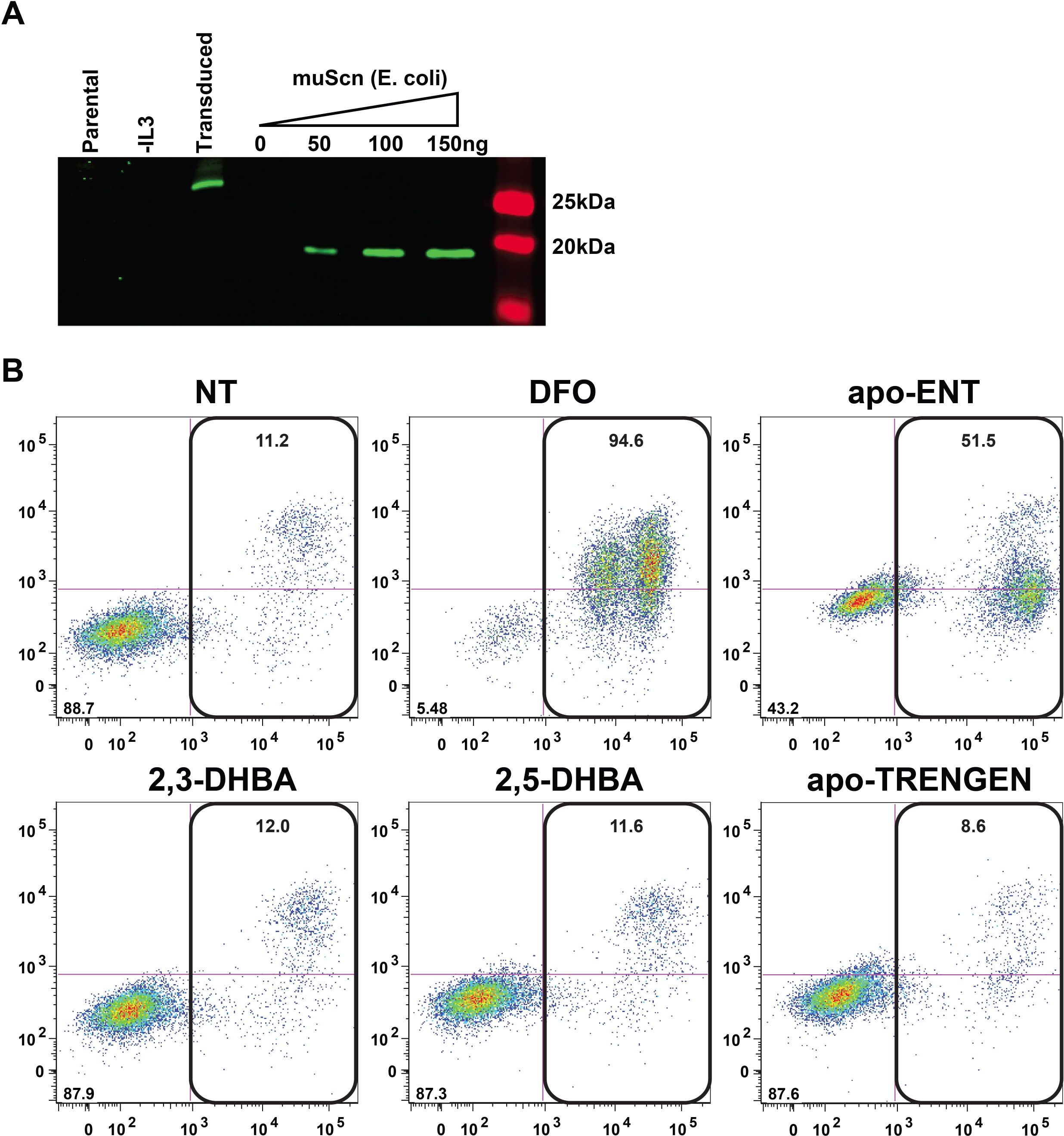

Supplement: Figure S4 — Related to Figure 7: Stably-induced expression of Scn does not drive apoptosis in 32D.3 cells. (A) A Western blot of 32D.3 cells shows that the transduced cells constitutively express Scn, while parental cells in the presence or absence of IL-3 do not secrete detectable amounts of Scn after 72 h in culture; 32 µL of culture supernatants was concentrated and loaded in the first three lanes. (B) Transduced 32D.3 were incubated with a variety of siderophores in order to assess the role of exogenous siderophores on cell viability. The hexadentate chelators DFO and Ent at 100 µM produce robust apoptosis, while the bidentate chelators at 300 µM do not affect viability. (TIF) [file pone.0043696.s004.tif]
