# Supplementary material for: Siderocalin/Lcn2/NGAL/24p3 Does Not Drive Apoptosis Through Gentisic Acid Mediated Iron Withdrawal in Hematopoietic Cell Lines
Source: PLoS One. 2012 Aug 21;7(8):e43696. doi: 10.1371/journal.pone.0043696 (PMC3424236; doi:10.1371/journal.pone.0043696)
Supplement: Table S1 — Related to Figure 8: Crystallographic statistics. (DOCX) [file pone.0043696.s005.docx]

**Supplemental Material: Table S1, related to Figure 8: Crystallographic statistics**

| **Data collection** |  |  |  |
| --- | --- | --- | --- |
| Structure | human Scn plus Fe(2,3DHBA)_3_ | human Scn plus iron/GA mixture | murine Scn/Fab complex |
| Space group | P2_1_2_1_2_1_ | P2_1_2_1_2_1_ | P2_1_2_1_2_1_ |
| Lattice constants (Å) | a = 117.48 b= 116.45 c = 120.91 | a = 115.42 b= 115.58 c = 119.86 | a = 82.13 b= 124.09 c = 147.49 |
| Resolution (Å) | 42.68-2.50 (2.60-2.50) | 41.60-2.90 (3.00-2.90) | 50.00-2.8 (2.90-2.80) |
| Unique reflections | 27696 | 35977 | 35241 |
| Average redundancy | 6.2 (4.86) | 5.44 (5.53) | 4.6 (4.4) |
| Completeness (%) | 94.2 (94.4) | 99.2 (99.9) | 93.1 (95.8) |
| R_merge_ (%) | 11.8 (37.5) | 7.1 (46.5) | 7.1 (36.1) |
| I/σ(I) | 11.0 (3.3) | 11.0 (2.3) | 19.8 (4.1) |
| **Refinement statistics** |  |  |  |
| R_work_ (%) | 24.7 | NA | 22.1 |
| R_free_ (%) | 28.8 |  | 28.9 |
| Number of atoms |  |  |  |
| Protein | 5550 |  | 8278 |
| FeDHBA | 139 |  | NA |
| Water | 184 |  | 61 |
| R.M.S deviations |  |  |  |
| Bond lengths (Å) | 0.012 |  | 0.009 |
| Bond angles (°) | 1.481 |  | 1.284 |
| Ramachandran |  |  |  |
| Favored (%) | 96.0 |  | 87.1 |
| Allowed (%) | 7.0 |  | 11.6 |
| Generously allowed (%) | 0.0 |  | 0.5 |
| Disallowed (%) | 0.0 |  | 0.7 |

Values in parentheses are for the highest resolution shell; the iron/GA complex was not refined, so no refinement statistics are provided; NA: not applicable.
