# Supplementary material for: Siderocalin/Lcn2/NGAL/24p3 Does Not Drive Apoptosis Through Gentisic Acid Mediated Iron Withdrawal in Hematopoietic Cell Lines
Source: PLoS One. 2012 Aug 21;7(8):e43696. doi: 10.1371/journal.pone.0043696 (PMC3424236; doi:10.1371/journal.pone.0043696)
Supplement: Experimental Procedures S1 — TRENGEN synthesis. (DOCX) [file pone.0043696.s006.docx]

**Supplemental Material: TRENGEN synthesis**

2,5-Bis-benzyloxy-benzoic acid benzyl ester (compound **2**; Figure S1): GA (compound **1**; Figure S1) (64.8 mmol) was dissolved in 500 mL acetone. Under Finkelstein reaction conditions, 3 equivalents of KI (195 mmol) and 7 equivalents of K_2_CO_3_ (454 mmol) were added to the reaction mixture. The reaction mixture was clear yellow with white, insoluble salts. Upon addition of benzyl chloride (195 mmol), the solution turned clear brown. The reaction mixture was refluxed in a mineral oil bath under a nitrogen atmosphere. After 72 h, the reaction was cooled to room temperature and the salts were filtered off. The filtrate was evaporated to be a white solid, after which it was redissolved in 200 mL methylene chloride and washed (2x150 mL) deionized water. The clear yellow solution was concentrated and loaded onto a silica column that was packed in 100% hexanes. The product was eluted with 100% methylene chloride. The fractions containing the product were collected and evaporated. The product was a white solid that was obtained in 87% yield. ^1^H NMR (500 MHz, CDCl_3_): δ 7.53 (s, 1H, Ar*H*), 7.46 (m, 10H, Ar*H*), 7.10 (d, 1H, Ar*H*, J =10 Hz), 6.98 (d, 1H, Ar*H*, J = 10Hz), 5.25 (s, 2H, PhC*H_2_*) 5.06 (s, 2H, PhC*H_2_*).^13^C NMR (75 MHz, CDCl_3_): δ 166.04, 152.73, 152.54, 136.92, 136.77, 136.03, 128.65, 128.56, 128.53, 128.28, 128.13, 128.10, 127.82, 127.60, 127.26, 127.26, 121.49, 120.40, 117.49, 116.03, 71.75, 70.73, 66.88. Elemental analysis: % C, H: estimated 79.22, 5.70, found 79.21, 5.73.

2,5-Bis-benzyloxy-benzoic acid (compound **3**; Figure S1): To a 500 mL roundbottom flask, **2** (56.2 mmol) was dissolved in 275 mL of methylene chloride. The solution was clear and colorless. A methanolic solution of KOH (1M, 100 mL MeOH) was added via addition funnel over the course of 20 minutes over air. Upon addition, the solution began to turn clear and yellow. The reaction stirred overnight and was monitored by TLC. KOH pellets were added to drive the reaction to completion as monitored by disappearance of the starting material spot in TLC. The solvent was evaporated, and the resulting mixture (light orange slurry) was dissolved in 200 mL deionized water. The reaction flask was cooled in an ice-water bath, and concentrated HCl (12M) was added dropwise to the stirring reaction mixture (clear, colorless solution) over air until the solution turned opaque and white. The white solid was filtered and dried *in vacuo* for 10 h (97% yield). ^1^H NMR (500 MHz, CDCl_3_): δ 7.84 (d, 1H, Ar*H*, J = 5 Hz), 7.37 (m, 15H, Ar*H*), 7.10 (dd, 1H, Ar*H*, J = 5 Hz, J= 10 Hz), 7.00 (d, 1H, Ar*H*, J = 10 Hz), 5.39 (s, 2H, PhC*H_2_*) 5.14 (s, 2H, PhC*H_2_*) 5.07 (s, 2H, PhC*H_2_*).^13^C NMR (75 MHz, CDCl_3_): δ 165.22, 153.75, 151.63, 136.55, 129.25, 129.19, 128.68, 128.21, 128.03, 127.63, 122.86, 117.58, 114.88, 72.98, 70.69. (+)FABMS: m/z calc. 355, obs. 355 (MH^+^).

(2,5-Bis-benzyloxy-phenyl)-(2-thioxo-thiazolidin-3-yl)-methanone (compound **4**; Figure S1): The white solid **3** (55.3 mmol) was dried *in vacuo* until powdery, then was dissolved in 175 mL benzene to give a clear, colorless solution. *N*-*N*’-dicyclohexylcarbodiimide (DCC) (60.9 mmol) was added to the solution, resulting in a translucent yellow solution. The addition of 2-mercaptothiazoline (55.3 mmol) over air and a catalytic amount of dimethylaminopyridine (0.17 mmol) resulted in an opaque, bold yellow solution. The reaction was stirred under a nitrogen atmosphere overnight, after which the excess DCC was filtered. The benzene was evaporated by rotary evaporation and the reaction mixture was a clear, orange oil that was soluble in methylene chloride. The mixture was loaded onto a silica column packed with 70:30 hexanes:methylene chloride. The product was eluted with 100% methylene chloride (R_f_ = 0.731 in 100% methylene chloride). ^1^H NMR (400 MHz, CDCl_3_): δ 7.44 (m, 10H, Ar*H*), 7.10 (m, 2H, Ar*H*), 6.88 (d, 1H, Ar*H*, J = 10 Hz), 5.09 (s, 1H, PhC*H_2_*), 4.98 (s, 2H, PhC*H_2_*) 4.48 (t, 2H, RNC*H_2_*, J = 10 Hz), 2.90 (t, 2H, RNC*H_2_*, J = 5 Hz). (+)FABMS: m/z calc. 436, obs. 436(MH^+^). Elemental analysis: % C, H, N: estimated 66.18, 4.86, 3.22, found 66.34, 5.06, 3.52.

TRENGEN-Bn_6_ (compound **5**; Figure S1): A 200 mL (18.08 g, 41.5 mmol) solution of **4** in methylene chloride (clear, yellow) was added to a 5 mL solution of triethylamine (2.07 mL, 13.8 mmol) dissolved in methylene chloride under a nitrogen atmosphere over the course of 5 minutes. The reaction was stirred overnight. A mixture of *mono-*, *bis-* and *tris-*substituted TREN compounds were obtained (In 4% MeOH: 96% methylene chloride TLC solution (R_f_ *mono*-TREN = 0.27, R_f_ *bis*- = 0.38, R_f_ *tris*- = 0.54; *tris*-substituted compound is visible by long-wavelength UV light). A silica column was packed in 100% methylene chloride and the product was eluted with 4% methanol to elute the *tris-*TREN substituted compound. ^1^H NMR (500 MHz, CDCl_3_): δ 7.98 (m, 3H, ArCON*H*), 7.85 (d, 3H, Ar*H*, J = 3 Hz), 7.34 (m, 30H, Ar*H*), 6.96 (dd, 3H, Ar*H*_,_ J_1_ = 3.5 Hz, J_2_ = 5.5), 6.69 (d, 3H, Ar*H*, J = 9 Hz), 5.11 (s, 6H, PhC*H_2_*), 4.87 (s, 6H, PhC*H_2_*), 3.25 (m, 6H, R_2_NC*H_2_*CH_2_NHCOAr), 2.30 (m, 6H, R_2_NCH_2_C*H_2_*NHCOAr). ^13^C NMR (75 MHz, CDCl_3_): δ 164.81, 153.01, 151.10, 137.01, 135.91, 128.81, 128.62, 128.03, 127.92, 127.63, 122.72, 119.93, 116.59, 114.65, 71.86, 70.50, 53.48, 51.91, 36.98.

TRENGEN (compound **6**; Figure S1): TRENGEN-Bn6 was placed in an EDTA-washed Schlenk flask. The flask was immersed in an ice-water bath and a 25 mL acid solution of 50:50 acetic acid:HCl was added dropwise. The reaction mixture was stirred for 48 h, or until the reaction was completed as assayed by thin layer chromatography. After the deprotection was completed, the acid was evaporated and subsequently coevaporated with methanol washes (5x10 mL MeOH) in vacuo until an off-white powder was obtained. R_f_ = 0.20 in 96:4 CH_2_Cl_2_:MeOH TLC solvent system. ^1^H NMR (500 MHz, CD_3_OD): δ 3.66 (t, 6H, NC*H*_2_CH_2_NCO, J_1_ = 42 Hz, J­_2_ = 11 Hz), 3.87 (t, 6H, NCH_2_C*H*_2_NCO, J­_1_ = 11 Hz, J_2_ = 42 Hz), 6.76 (d, 3H, Ar*H*, J = 8.2 Hz), 6.87 (d, 3H, Ar*H*, J = 2.5 Hz), 7.21 (s, 3H, Ar*H*). ESI (+) 555.21 m/z (M+H). IR (ZnSe crystal, neat): *v* 1488, 1557, 1646, 2360, 3277 cm^-1^. Elemental analysis for TRENGEN•Cl•CH_3_COO^-^: % C, H, N: estimated 47.30, 4.69, 6.69, found 47.52, 4.70, 6.83.
